# Context-dependent actions of STING pathway in colitis and associated colon cancer

**DOI:** 10.1016/j.gendis.2025.101855

**Published:** 2025-09-18

**Authors:** Jiaorong Qu, Yajie Cai, Fanghong Li, Yufei Li, Runping Liu

**Affiliations:** a School of Life Sciences, Beijing University of Chinese Medicine, Beijing 100029, China; b School of Chinese Materia Medica, Beijing University of Chinese Medicine, Beijing 100029, China

**Keywords:** cGAS, Colitis, Colon cancer, Innate immunity, STING

## Abstract

Inflammatory bowel disease (IBD), a prevalent chronic inflammatory disorder with unsatisfactory therapeutic outcomes, significantly increases the risk of colorectal cancer. The cyclic GMP-AMP synthase (cGAS) and stimulator of interferon gene (STING), highly expressed in human IBD, are potential anti-inflammatory and anti-tumor immunotherapeutic targets. However, conflicting evidence regarding the dual roles of the STING pathway has significantly hindered its development as a therapeutic target for innovative treatments. Previous studies have predominantly suggested that hyperactivation of the STING pathway contributes to colitis development, while simultaneously enhancing anti-tumor immunity and inhibiting cancer progression. On the other hand, specific contexts, such as STING deficiency in T cells or prolonged, excessive STING activation within tumors, paradoxically promote disease progression. We also thoroughly analyzed the origin of STING activation in these diseases to offer insights into the identification of novel druggable targets. Crucially, “cell context-dependency, treatment timing and duration, and biased signal transduction” are likely the mechanistic basis underlying STING pathway’s dual roles, proposing spatiotemporal-specific STING modulators as future therapeutics.

## Introduction

Inflammatory bowel disease (IBD), encompassing Crohn’s disease and ulcerative colitis, is characterized by chronic gastrointestinal inflammation, severe symptoms (*e.g.*, bloody diarrhea, abdominal pain), and diminished quality of life.^1^ Given the high prevalence and severe symptoms, such as bloody diarrhea, abdominal pain, urgency, and tenesmus, ulcerative colitis generally receives more attention than Crohn’s disease.^2^ Although the annual incidence and prevalence of IBD are rapidly rising globally, especially in Asia, Africa, and the Middle East, the precise etiopathogenesis of IBD remains limited.^3^ Dysregulated innate/adaptive immunity, microbial dysbiosis, and genetic susceptibility loci (*e.g.*, cytokine/chemokine regulators identified via GWAS) collectively drive IBD progression.^4^ Notably, IBD patients exhibit a 2-to-3-fold increase in risk of colon cancer compared with normal people, which ranks among the top three malignancies worldwide, with 1.2 million annual cases and 600,000 deaths. Alarmingly, colon cancer incidence is rising in individuals under 50. Recent in-depth studies have identified significant characteristics of inflammation-to-cancer transformation in colon cancer.^5^ Mechanistically, chronic inflammation promotes carcinogenesis via three pathways: i) DNA damage (reactive oxygen species/reactive nitrogen intermediates-induced mutations, *p53* inactivation, epigenetic changes); ii) immune dysregulation (pro-inflammatory cytokines enhancing tumor proliferation/apoptosis resistance); and iii) microbiota-derived metabolites. Targeting the inflammatory microenvironment may thus mitigate both colitis and cancer progression.

In clinical practice, 5-aminosalicylate remains the first-line pharmacological intervention for patients with mild-to-moderate IBD. The chemical therapies of IBD is upgraded on top of 5-aminosalicylate according to the severity of the disorder, including corticosteroids, immunomodulator (*e.g.*, thiopurines, cyclosporine, and azathioprine), tumor necrosis factor (TNF) inhibitors (*e.g.*, infliximab and adalimumab), adhesion inhibitors (*e.g.*, vedolizumab), interleukin (IL)-12/IL-23p40 inhibitors (*e.g.*, ustekinumab), and Janus kinase (JAK) inhibitors.^2^^,^^6^^,^^7^ However, incomplete remission, relapse, and adverse effects (pancreatitis, hepatitis, lymphoma risk) underscore unmet therapeutic needs. For colon cancers, surgery as well as neoadjuvant therapy (*e.g.*, short-course and long-course radiotherapy) are the major therapeutic methods, often combined with other adjuvant chemotherapies (*e.g.*, fluorouracil-containing regimens and oxaliplatin). Perioperative immunotherapy, encompassing both neoadjuvant and adjuvant approaches, has garnered increasing attention in recent years.^8^ Neoadjuvant immunotherapy, particularly prominent since 2020, activates anti-tumor immunity to shrink tumors and facilitate surgical resection in advanced cases, while adjuvant immunotherapy sustains immune surveillance to reduce postoperative recurrence and metastasis. However, recurrence and treatment resistance remain significant challenges. Therefore, early intervention targeting the inflammation stage of pre-malignancy may offer greater efficacy.

As reported, the cyclic GMP-AMP (cGAMP) synthase (cGAS)–stimulator of interferon genes (STING) signaling pathway, implicated in both inflammatory progression and anti-tumor immunity, has emerged as a pivotal regulator in IBD and colon cancer.^6^^,^^9^^,^^10^ In colitis, epithelial damage triggers cytosolic accumulation of aberrant dsDNA (including mitochondrial and nuclear DNA) and exosomal dsDNA release,^11^ while gut-derived bacterial cyclic dinucleotides (CDNs) further activate STING signaling to amplify inflammation.^12^ In the pathogenesis of colon cancer, deficiencies in DNA damage repair, accumulation of damaged DNA, and bacterial-derived dsDNA have also been identified as key signaling sources for the activation of the cGAS–STING pathway.^13^ Consequently, the cGAS–STING pathway may exhibit heightened sensitivity in these colon diseases. This review synthesizes the mechanistic duality of STING in the progression of colitis and colitis-associated cancer, providing a framework for targeting this pathway in inflammation-driven malignancies.

## The molecular basis of cGAS–STING signaling pathway

The cGAS–STING pathway is triggered by endogenous or exogenous DNA, which distinguishes it from other innate immune defense pathways. Inactive cGAS maintains autoinhibition until dsDNA binding induces dimerization. cGAS dimers bind to dsDNA asymmetrically via their C-terminal nucleotidyltransferase domains, forming a 2:2 cGAS-DNA complex through phosphate backbone interactions. Longer stretches of dsDNA molecules offer a more stable network structure with cGAS, thus enhancing the catalytic activity of cGAS to cyclize cGAMP.^7^ The synthesized cGAMP activates endoplasmic reticulum (ER)-resident STING, inducing dimer oligomerization and ER-to-Golgi trafficking via ER-Golgi intermediate compartment (ERGIC). At the Golgi, STING recruits TANK-binding kinase 1 (TBK1), which phosphorylates its C-terminal tail (Ser366) and interferon regulatory factor 3 (IRF3), triggering IRF3 dimerization, nuclear translocation, and type I interferon (IFN)/interferon-stimulated gene (ISG) induction. Concurrently, STING activates inhibitory kappa B kinase (IKK) to activate nuclear factor kappa B (NF-κB)-dependent cytokine production.^14^ Palmitoylation of STING cysteine residues mediates TBK1 recruitment and signaling amplification. Post-activation, lysosomal degradation of STING prevents hyperactivation, establishing a critical feedback loop.^15^

## The dual function of the STING pathway in colitis

### Inhibiting STING activation protects against colitis

cGAS–STING has emerged as a pro-inflammation and pathogenetic pathway in the initiation of colitis by directly modulating the secretion of proinflammatory cytokines, including those from the interleukin family and TNF family. Clinically, higher phosphorylation levels of STING and following IRF3 activation were detected in the macrophages of colonic mucosal tissue in patients with active Crohn’s disease when compared with control patients.^11^ The single-cell RNA-sequencing dataset also suggested that cGAS, STING, TBK1, IRF3, type I IFNs, and downstream ISGs were increased in colonic mucosal biopsy tissues from patients with active ulcerative colitis.^16^ Liraz et al observed increased protein levels of STING, rather than the transcription of STING, in multiple acute and chronic experimental colitis mouse models induced by *Salmonella enterica serovar*, *Citrobacter rodentium*, dextran sulfate (DSS), and T cell adoptive transfer.^17^ A previous study utilized STING-mutant C57BL/6J mice carrying a missense mutation in exon 6 of the transmembrane protein 173 (Tmem173) gene. The STING-mutant mice were incapable of producing IFN-β in response to CDNs or *Listeria monocytogenes* infection. In the mouse model of DSS induction, STING-mutant mice showed relieved colitis severity, while STING agonists 5,6-dimethylxanthenone-4-acetic acid (DMXAA) greatly worsen the intestinal inflammation in the wild-type (WT) mice.^12^ Mechanistically, *in vitro* experiments indicated that the elevated STING expression was observed in M1-polarized macrophages derived from murine and human, and it also induced M2-polarized macrophages to transform into an M1-like subtype.^12^ NLR family pyrin domain containing 3 (NLRP3) deficiency aggravated radiation-induced colitis by enhancing epithelial barrier disruption and activating STING signaling (increased TBK1/IRF3 phosphorylation and IFN-β production in bone marrow-derived macrophages).^18^ Li et al demonstrated that 2,3-dioleic acid containing epoxy groups increased the intestinal permeability induced by DSS via activating cGAS–STING/myeloid differentiation factor 88 (Myd88)/NF-κB signaling, thereby releasing inflammatory cytokines, including IL-6, TNF-α, IL-1β, and IFN-γ.^19^

Moreover, a mouse model with heterozygous expression of the STING genetic variant N153S, which showed constitutive activation of STING independent of cGAMP due to the gain-of-function mutations in Tmem173, was utilized to investigate the impact of the hyperactivation of the STING pathway on colonic inflammation. Constitutive activation of STING in N153S mice also promoted spontaneous chronic colitis and fibrosis, which was dependent on the accumulation of CD4^+^ T cells and was not linked with the activation of STING in intestinal epithelial cells. Moreover, severe intestinal inflammation with STING accumulation was developed in WT mice reconstituted with bone marrow cells from N153S mice, suggesting that STING in myeloid cells was the initial driver of colitis. In addition, K63-linked ubiquitination-mediated STING stabilization was detected in cluster of differentiation 11B-positive (CD11b^+^) myeloid cells from N153S mice under conditions of intestinal inflammation.^17^ In our recent investigation, myeloid STING knockout in adult mice dramatically ameliorated DSS-induced colitis through limiting dendritic cell activation, suppressing macrophage maturation, and inhibiting the differentiation of Th1 and Th17 cells. The *in vitro* experiments showed that the deletion of STING resulted in suppressed secretion of IL12 family cytokines in primary bone marrow-derived macrophages and bone marrow-derived dendritic cells, thus obstructing the differentiation of splenic Th1 and Th17 ^20^. Furthermore, in a murine model of colitis induced by deletion of IL-10, the absence of cGAS and STING both reversed intestinal inflammations, as evidenced by the reduction of proinflammatory cytokines, including IL-1β, IL-22, and IL-12 ^21^.

### Inhibiting STING activation aggravates the development of colitis

**Figure 1:**
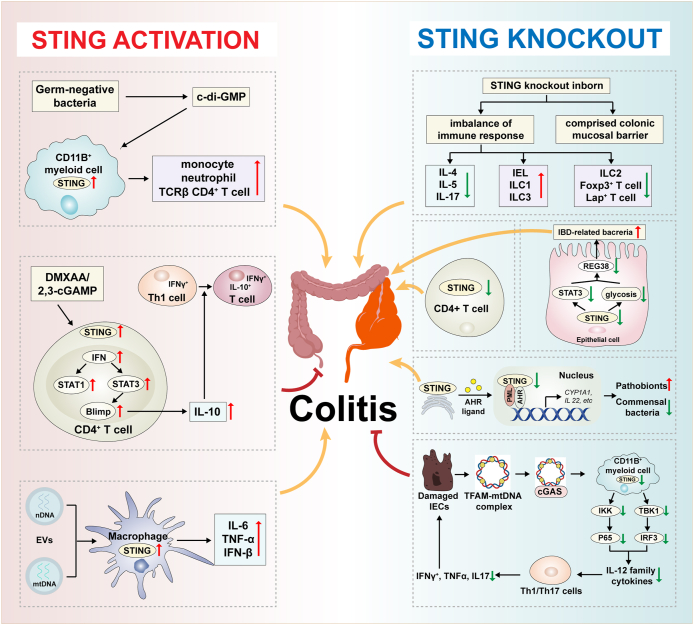
Controversial effects of the STING pathway in colitis development. The activation of the STING pathway in CD11b^+^ myeloid cells and macrophages plays a pathogenic role in the development of colitis by up-regulating specific immune cells and pro-inflammatory factors. The knockout of the STING pathway in Lysm^+^ cells ameliorates colonic inflammation through obstructing the differentiation of Th1 and Th17 cells via suppressing IL-12 family cytokines. However, STING deficiency in epithelial cells and CD4^+^ T cells aggravates intestinal inflammation, while STING activation in CD4^+^ T cells exerts a remission role in colitis pathogenesis. The absence of nuclear STING inhibits AHR activity to exacerbate colitis due to intestinal microbiota dysbiosis. Furthermore, congenital absence of STING contributes to the compromised colonic barrier and imbalance of immune responses.

Although the pathogenic effect of the STING pathway in colitis has been determined by an overwhelming majority of studies, some reports have suggested that STING activation in turn exerts a remission role in the pathogenesis of colitis (Fig. 1; Table 1). Yang et al recently found that STING knockout exacerbated DSS-induced acute colitis, while deficiency of STING in CD4^+^ T cells induced more severe colonic inflammation independent of intestinal microbiota. Notably, the elevated IL-10 production induced by the transplantation of CD4^+^ T cells derived from patients with ulcerative colitis or Crohn’s disease was further dose-dependently induced by 2,3-cGAMP in mice. STING activation induced the translocation of IRF3 into the nucleus and mitochondria, leading to a decrease in the population of pathogenic Th1 cells and the production of the anti-inflammatory cytokine IL-10. The expression of key transcription factors related to the up-regulation of IL-10, B-lymphocyte-induced maturation protein 1 (Blimp), and mitochondrial oxidation was also increased following STING activation.^22^ Similarly, Canesso et al found that congenital absence of STING contributed to a lower goblet cell number, decreased mucus production, and reduced secretory IgA. The down-regulation of intraepithelial lymphocytes and group 2 innate lymphoid cells (ILC2), as well as elevated frequencies of ILC1 and ILC3 in the colon, were observed due to the absence of STING. The function of forkhead box P3-positive (Foxp3^+^) and latency-associated peptide-positive (LAP^+^) regulatory T cells was also impaired.^23^ Similarly, in our recent report, neonatal deletion of myeloid STING induced severe colitis with reduced infiltration of CD11b^+^ cells, probably indicating that STING knockout in neonatal mice might disrupt immune tolerance in the colon by interfering with the maturation of myeloid lineage immune cells.^20^ Moreover, STING-deficient mice were more susceptible to enteric infection with *Citrobacter rodentium* when compared with WT mice, as characterized by severe intestinal inflammation and impaired bacterial clearance ability. Mechanistically, STING suppresses the signal transducer and activator of transcription 3 (STAT3) activation and inhibits glycolysis to reduce regenerating islet-derived protein 3γ (REG3γ) production in intestinal epithelial cells, thus hindering the clearance of IBD-related bacteria.^24^ In addition, a most recent study reported that nuclear STING1 promoted transcription activity of aryl hydrocarbon receptor (AHR) independently of the canonical cGAS–STING1–IRF3 pathway through recruiting the transcriptional coactivator PML nuclear body scaffold (PML). Deficiency of STING abolished the AHR ligand ITE-regulated protective effect against DSS-induced colitis. Furthermore, STING1 was indispensable in the AHR-mediated microbial regulation against DSS treatment, especially in *Lactobacillus* and *Helicobacter*.^25^

**Table 1:** The effects of STING on the progression of colitis.

Number	The function on STING	Mouse model	Triggers	Downstream signaling	Target cell	Reference
*Aggravate colitis*						
1	STING knockout	DSS-induced colitis	/	Down-regulation of IEL, ILC2, Foxp3^+^ and LAP^+^ regulatory T cells, and up-regulation of ILC1 and ILC3	/	23
2	STING knockout	*Citrobacter rodentium*-induced colitis	/	STAT3/REG3γ	/	24
3	STING knockout	DSS-induced colitis	Extracellular vesicle-encapsulated dsDNA	IRF3 & NF-κB	/	11
4	STING-mutant	DSS-induced colitis	/	PML/AHR	Macrophage	25
5	STING activation	Radiation-induced colon damage	/	TBK1/IRF3/IFN-β	Macrophage	18
6	STING activation	DSS-induced colitis	/	Myd88/NF-κB	Intestinal epithelial cell	19
7	STING activation	DSS- or TNBS-induced colitis	Genomic instability, DNA damage	IRF3 & NF-κB	Intestinal stem cell	27
8	Constitutive activation of STING	*Salmonella enterica serovar*-, *Citrobacter rodentium*-, DSS-, and T cell adoptive transfer-induced colitis	Bacterial cyclic di-GMP	IL-1β, RANTES	Myeloid cell	17
9	Global and CD4^+^ T cell-specific STING knockout	DSS-induced colitis	/	Blimp1/type I IFN/STAT3	CD4^+^ T cell	22
*Alleviate colitis*						
1	STING knockout	DSS-induced colitis	Microbiota-derived c-di-AMP & c-di-GMP	MyD88/IL-1β & IL-18	Monocyte	21
2	STING-mutant	DSS-induced colitis	Mammalian 2′,3′ -cGAMP/bacterial c-di-AMP	/	Macrophage	12
3	Meloid STING knockout	DSS-induced colitis	Damaged epithelial cell-derived TFAM-associated mtDNA	TBK1/IRF3/IL12 & NF-κB/12	Dendritic cell and macrophage	20

Note: IEL, intraepithelial lymphocytes; DSS, dextran sulfate; TNBS, 2,4,6-trinitrobenzene sulfonic acid; ILC1/2/3, group 1/2/3 innate lymphoid cell.

Based on the above evidence, the reason for the dual function of STING may be that the downstream immune events mediated by STING pathway activation in different types of immune cells, or even different subcellular structures, are distinct. Thus, to fully investigate the specific functions and mechanisms of the STING pathway in specific cell types, more detailed lineage-specific knockout is needed, rather than just using systemic knockout animals. Manipulating the subcellular translocation of STING through genetic approaches (the addition or removal of a localization signal peptide) could also provide deeper insights into how non-canonical STING activation signals suppress the development of IBD.

### Tracing the trigger of STING activation in colitis

Recently, extracellular vesicle (EV)-encapsulated dsDNA, genomic instability, and commensal intestinal bacterium have been found to be the vital and independent triggers of the STING pathway activation during the development of colitis. EVs isolated from the plasma of Crohn’s disease patients or lipopolysaccharide-stimulated murine colonic epithelial cells initiated the activation of the STING pathway in macrophages. Specifically, the inhibition of EV release by GW4869 treatment in murine colitis significantly lowered the level of exosomal dsDNA, suppressed STING activation, and alleviated inflammatory responses, indicating that the transmission of pathogenic DNA signaling is EV-dependent.^11^ However, whether these dsDNA-containing exosomes originated from intracellular or extracellular domains has not been elucidated clearly. EV-free dsDNA derived from intrinsic dead cells and extracellular viruses, which were not dependent on EV secretion, were also illuminated to play a crucial role in triggering the activation of the cGAS–STING pathway in inflammatory diseases, which remained to be investigated at the onset of colitis.^26^ Therefore, in our recent *in vitro* experiments, we utilized EV release inhibitor GW4869, DNase, RNase, and endocytosis inhibitor dynasore to figure out the origin of STING activation in colitis. We found that mitochondrial transcription factor A (TFAM)-associated mtDNA released from damaged intestinal epithelial cells, rather than RNA and EVs, was taken up by bone marrow-derived dendritic cells through endocytosis to activate the STING pathway and downstream IL-12 family cytokines in bone marrow-derived dendritic cells.^20^ Moreover, genomic instability mediated by cGAS–STING pathway was found in the pathogenesis of colitis. Ren et al demonstrated that deficiency of epithelial DEAH-box helicase 9 (DHX9) in mice (referred to as *Dhx9*^ΔIEC^ mice) caused abnormal accumulation of DNA:RNA hybrids (R-loops), subsequently triggering genomic instability. Blocking the cGAS–STING pathway in *Dhx9*^ΔIEC^ mice partially ameliorated the colitis phenotype and partially restored the proportions of intestinal stem cells and goblet cells. This result indicated that the activation of cGAS–STING pathway amplified inflammatory responses in colitis, attributed to DHX9 deficiency-induced DNA damage.^27^

On the other hand, growing experimental evidence concluded from intestinal bacteria and fecal microbiota transplantation experiments strongly suggested that dysbiosis functioned as a predominant driver of intestinal inflammation in mice with constitutive activation of STING.^17^ N153S mice with colitis phenotypes displayed a significant increase in the abundance of microbiota associated with IBD when compared with WT mice, including *Enterobacteriaceae*, *Helicobacteraceae*, *Lactobacillaceae*, and *Peptostreptococcaceae*. In addition, the relative abundance of potential probiotics, such as *Lachnospiraceae* and *Rikenellaceae*, was reduced in N153S mice.^17^ Jeonghyun et al found that STING-deficient mice exhibited a slight reduction in body weight in response to acute DSS treatment compared with WT mice. However, the difference in body weight between STING knockout and WT mice under the administration of DSS was not significant when *Helicobacter* spp was absent.^21^ The elimination of *Helicobacter typholonlus* by neomycin inhibited STING expression and relieved inflammation in N153S mice.^17^ A broad-spectrum antibiotic cocktail that mostly targets Gram-negative bacteria could also rescue colitis-related disease manifestations, suggesting that Gram-negative bacteria might play a more significant role in STING activation. Furthermore, bacterial CDNs, such as cyclic di-GMP, directly secreted by bacteria, enhanced the stabilization of STING by inducing K63-linked ubiquitination in bone marrow-derived macrophages from N153S mice.^17^ Also, pro-inflammatory cytokines, including IL-1β and IL-18, were reduced in CDN-treated *STING*^*−/−*^ bone marrow-derived macrophages and bone marrow-derived dendritic cells, indicating that CDNs served as the driver of colitis-associated inflammatory cytokine secretion dependent on STING.^21^ Notably, the amelioration of colitis was more significant in cGAS-depleted mice compared with those with STING deficiency, implying that the sensing of CDNs by cGAS is essential for the activation of the STING pathway.^21^ Collectively, these results highlight that gram-negative bacteria-derived products are unneglectable triggers of cGAS–STING activation in colitis. Relying on fecal microbiota transplantation or probiotic colonization to competitively outcompete these CDN-producing and potentially pro-inflammatory bacteria may serve as an ideal adjunctive therapy for colitis.

## The dual function of STING signaling in colon cancer

### The anti-tumor effects of STING activation mediated by IFN-dependent pathway

Contrary to the harmful regulatory effects demonstrated in colitis, the STING activation displayed an anti-tumor effect via remodeling the tumor immune microenvironment and was gradually considered as an independent prognostic factor in colon cancer. Yao et al reported that STING expression was up-regulated in cancer tissues compared with adjacent normal tissues in the thirty-two paired colorectal cancer and adjacent normal tissues.^28^ Tian et al found that increased STING mRNA and protein levels were related to better survival rate and better 5-fluorouracil-associated chemotherapy response in 58 specimens of The Cancer Genome Atlas (TCGA) colon adenocarcinoma patients.^29^ Another report assessed endothelial STING levels in tumor tissues from 160 patients with colon cancer. High endothelial STING expression was significantly associated with increased CD8^+^ T cell infiltration and prolonged the overall survival.^30^^,^^31^ Recently, Kuanc et al randomly collected 41 biopsy samples from stage IV colon cancer patients, including 20 patients with microsatellite stable tumors and 21 patients with microsatellite instability-high tumors. Increased levels of cGAS and STING were positively related to microsatellite instability-high stage-IV colon cancer and associated with prolonged survival, good prognostic effects, and good immunotherapy responses.^32^ Interestingly, according to the RNA-sequencing data of 26 paired colon cancer tissues and adjacent non-tumor tissues from the TCGA database, the expression variations of STING and cGAS were consistent only in a few patients with stage I or II colon cancer. In most cases, both elevated cGAS and reduced STING expression were observed in the tumor samples when compared with the adjacent normal samples of the same patient, suggesting that the interruption of the cGAS–STING pathway occurred in patients with colon cancer.^33^ Insufficient STING activation in cancer patients suggested the need to activate STING for anti-tumor effects. Furthermore, single-nucleotide polymorphisms of STING and IFNB1 were explored as effective predictors of the therapeutic effect of cetuximab in patients with metastatic colon cancer. To be specific, patients carrying the variants of STING rs1131769, IFNB1 rs1051922 G/A, and A/A genotype exhibited markedly shorter overall survival under the treatment of cetuximab.^34^ As the downstream effectors of the cGAS–STING pathway, IFNs secreted by immune cells are responsible for tumor-associated antigen presentation and regression in tumor models, subsequently inhibiting the tumor process. Hence, stimulating cGAS–STING–IFN signaling might play a therapeutic role in colon cancer.^35^

In addition to clinical data, numerous studies have investigated the underlying mechanisms of STING in the development of colon cancer using animal models. It was noted that STING-deficient mice displayed higher susceptibility in the azoxymethane (AOM)/DSS-induced colon cancer mouse model, providing direct evidence for the protective role of STING signaling against colon cancer.^36^^,^^37^ Zhu et al reported that the absence of STING exacerbated inflammation 14 days post-AOM injection, which was manifested by elevated numbers of proliferative epithelial cells and increased expression of inflammatory cytokines, including IL-6 and keratinocyte chemoattractant (KC), in the colon and serum. Meanwhile, the phosphorylation of NF-κB and STAT3 was also promoted in STING-deficient mice during the early stages of tumor development.^36^ IL-18 and interleukin-22 binding protein (IL-22BP) are downstream factors of STING, and mice deficient in IL-18 or IL-22BP are highly susceptible to colon cancer. Ahn et al found that after AOM/DSS administration, STING knockout mice developed more tumors, associated with a significant reduction of IL18 and IL-22BP. Down-regulated IL-22BP was further proved to be a result of reduced IL-18 in STING-deficient bone marrow-derived macrophages, suggesting a potential anti-tumor mechanism involving the interplay between STING and IL-18 ^37^. These findings were further supported by a recent study. It was recognized that mice lacking spleen tyrosine kinase (SYK) were susceptible to IBD and colon cancers due to the deficiency in inflammasome assembly and gasdermin-mediated pyroptosis.^38^ Moreover, the phosphorylation of Tyr240 of STING, which is essential for STING-mediated IFN induction, is dependent on SYK activation.^39^ Also, gasdermin D (GSDMD)-mediated pyroptosis functioned as a negative regulation for the STING pathway during inflammation. Gong et al reported that STING activation, SYK phosphorylation, GSDMD cleavage, and the direct interaction between STING and SYK were observed in colonic tumor tissues from colon cancer patients. In the AOM/DSS mouse model, accompanied by decreased SYK phosphorylation and GSDMD cleavage, reduced expression of pyroptosis-related cytokines IL-1β and IL-18 was observed in the tumor of STING knockout mice. *In vitro* experiment further showed that STING activation induced by 2′3′-cGAMP promoted GSDMD cleavage via increasing SYK phosphorylation in HT-29 cells. This research indicated that STING activation could prevent tumorigenesis of colon cancer by inducing SYK-dependent cell pyroptosis.^40^

Additionally, Xia et al detected the expression of cGAS and STING in 11 colon cancer cell lines, finding that cGAS–STING signaling is defective in most cell lines, which is attributed to the epigenetic hypermethylation of cGAS.^41^ Vornholz et al applied retroviral particles to transduce dominant active STING variants (STING^N153S^) into MC38 cells to establish MC38 cell lines with sustained activation of STING. The hyperactivated STING-IFN signaling in STING^N153S^-transduced MC38 cells significantly blunted tumor growth in the xenograft model, accompanied by increased infiltration of cytotoxic T lymphocytes and natural killer (NK) cells, as well as enhanced expression of cytotoxic effector molecules, such as granzyme B (Gzmb), perforin 1, IFN-γ, and TNF, in the tumor microenvironment. Additionally, genetically enforced STING activation in MC38 cells was sufficient to enhance the efficacy of immune checkpoint inhibitor therapy. Compared with mice injected with pure WT MC38 cells, anti-PD1 and anti-cytotoxic T-lymphocyte antigen 4 (CTLA4) inhibitors achieved better efficacy in mice injected with a mixture of STING^N153S^-expressing and WT MC38 cells, as indicated by slower tumor growth and increased survival rate. Elevated frequencies of immune cells, including CD8^+^ cytotoxic T lymphocytes, as well as up-regulated immune factors, including IL12 and IFN-γ, were also detected in response to immune checkpoint inhibitor therapies. Cellular indexing of transcriptomes and epitopes by sequencing suggested that the activation of STING signaling reprogrammed immune microenvironments was related to multiple antigen-presenting lymphocytes in STING^N153S^-expressing tumors, including significant enrichment of macrophages, major histocompatibility complex II-positive (MHC II^+^) dendritic cells, CD11b^+^ dendritic cells, and plasmacytoid dendritic cells.^42^ To further explore the involvement of different innate immune cell lineages in STING-mediated colon cancer immune homeostasis, Ahn et al employed two types of mouse lines expressing Cre-recombinase in different mononuclear phagocytes. These two strains include *Tmem173*^*fl/fl*^*Lysm*^*Cre*^ mice, which eliminate STING in macrophages and neutrophils, and *Tmem173*^*fl/fl*^*Cd11c*^*Cre*^ mice, which eliminate STING from dendritic cells. In response to AOM/DSS induction, the body weight loss was improved and the polyp formation was reduced in both *Tmem173*^*fl/fl*^*Lysm*^*Cre*^ mice and *Tmem173*^*fl/fl*^*Cd11c*^*Cre*^ mice, when compared with WT mice and mice with global knockout of STING.^21^ Furthermore, we obtained *Tmem173*^*fl/fl*^*Lysm-Cre*^*ert2*^ mice to investigate the effects of myeloid STING knockout after tumor formation by performing tamoxifen induction after the induction of AOM/DSS. As expected, deletion of myeloid STING after tumor formation significantly facilitated tumor growth via inhibiting a series of innate and adaptive immune,^20^ suggesting that STING signaling in mononuclear phagocytes plays a crucial role in the progression of colon cancer. While it is plausible that the STING pathway in other cell lineages may play a role in maintaining gut immune homeostasis during carcinogenic events, there is currently a lack of critical evidence to confirm this.

Several studies have reported on additional pathways involved in STING-mediated anti-tumor effects, which may act independently of type I IFN responses. In MC38 cells, the knockout of IFN-α/β receptor (IFNAR), which is essential for the expression of a large group of ISGs in response to type I IFN, had no impact on tumor growth, indicating that the anti-tumor effect of intrinsic STING is probably independent of type I IFN responses in cancer cells.^43^ Sun et al reported that in both global and myeloid-specific STING knockout mice, more colon cancer liver metastatic lesions were found, and the cytotoxic properties of NK cells were inhibited. By investigating the communication between tumor-associated macrophages and NK cells, they found that the deletion of STING down-regulated the expression of 4-1BBL in macrophages and 4-1BB (CD137) in NK cells. Notably, the blockade of either 4-1BBL or 4-1BB significantly increased MC38 liver metastasis. The expression of IFN-γ was not implicated during this process, indicating that IFN-γ was not involved in the regulation of STING–4-1BBL/4-1BB signaling-mediated NK cell activation. On the other hand, IL-18 and IL-1β could synergistically cooperate with 4-1BBL/4-1BB signaling to stimulate NK cell activity, which is dependent on NLRP3 activation in macrophages. Specifically, myeloid NLRP3-deficient mice showed more severe tumor burden, since the expression of 4-1BBL in macrophages and 4-1BB in NK cells was inhibited. The administration of NLRP3 agonist, nigericin, abrogated colon cancer liver metastasis in mice with myeloid STING knockout by elevating IL-18 and IL-1β, as well as recovering NK cell activation.^31^ Additionally, in the development of colon cancer, ferroptosis was also strongly activated in response to STING signaling in an IFN-independent manner. Based on the analysis of the TCGA database, in colon cancer, STING was found to have a significant negative association with the expression of aldo-keto reductase family 1 member C1 (AKR1C1), a gene considered to be associated with ferroptosis-related risk and an unfavorable prognostic signature. Meanwhile, STING was significantly and positively co-expressed with farnesyl-diphosphate farnesyltransferase 1 (FDFT1) and ATP synthase membrane subunit C locus 3 (ATPS5MC3), both of which are ferroptosis-related protective genes associated with better prognosis in colon cancer patients. Aside from STING itself, research has also highlighted the preventative effect of cGAS on carcinogenesis, which appears to be independent of type I IFN responses. cGAS, but not STING, is highly expressed in the intestinal stem cell population. The deletion of cGAS leads to a series of pro-oncogenic effects, such as inducing intestinal stem cell loss, destroying intestinal barrier integrity, facilitating inflammatory phenotypes, and promoting the proliferation of tumor cells. Another report found that the absence of cGAS in both hematopoietic and non-hematopoietic cells accelerated tumor development. Meanwhile, mice lacking cGAS were reported to be more susceptible to AOM/DSS-induced colon cancer than those lacking either STING or type I IFN receptor.^44^ Mechanistically, cGAS deficiency induced the activation of STAT1 and STAT3 but not type I IFN signaling to promote the colon cancer process. Moreover, increased population of Th17 cells and accumulated tumor-promoting myeloid-derived suppressor cells were also found in mice lacking cGAS, which might be caused by up-regulated C-X-C motif chemokine ligand 1 (CXCL1) and C–C motif chemokine ligand 2 (CCL2), as well as decreased IL-10 production.^44^

## STING activation is potentially involved in the exhaustion of cytotoxic lymphocytes and radiotherapy resistance of colon cancer

Notably, sustained type I IFN expression in response to STING activation has recently been reported to exacerbate the exhaustion of cytotoxic CD8^+^ T cells, thus favoring the escape of cancer cells from immunosurveillance.^45^ Mechanistically, chronic stimulation by type I IFNs disrupts lipid metabolism and redox balance in CD8^+^ T cells, leading to a terminal exhausted phenotype, as characterized by positive programmed cell death protein 1 (PD-1), lymphocyte activation gene 3 (LAG3), and CTLA4 or PD-1, LAG3, CTLA4, T-cell immunoglobulin and mucin domain 3 (TIM3), and T-cell immunoreceptor with immunoglobulin and immunoreceptor tyrosine-based inhibitory motif domains (TIGIT) expression.^46^ Type I IFNs could also drive CD8^+^ T cell exhaustion via an IRF7-dependent mechanism, potentially bridging progenitor cells and exhausted cells within the IFN-stimulated subset.^47^ Similarly, in the MC38 colon cancer model, the transcription factor IRF2 is expressed by numerous immune cells within the tumor under sustained IFN signaling, and serves as a pivotal feedback molecule redirecting IFN signals to induce CD8^+^ T cell exhaustion, thus presenting a promising target for enhancing colon tumor control.^48^ Furthermore, cGAS–STING activation-induced type I IFNs releasing contributes to the establishment of an immunosuppressive tumor microenvironment through other immune cells. In several cancers, including oral squamous cell carcinoma and hepatocellular carcinoma, STING activation was involved in the up-regulation of programmed cell death ligand 1 (PD-L1) and PD-1.^49^ A most recent study suggested that the activation of the cGAS–STING pathway in macrophages induced IFNα synthesis, resulting in bone marrow stromal cell antigen 2 (BST2) overexpression in the tumor microenvironment of pancreatic ductal adenocarcinoma, and promoting malignant tumor progression. Extracellular signal-regulated kinase (ERK)–CXCL7 signaling was then found to be critical for the BST2 expression-regulated CD8^+^ T cell exhaustion.^50^ Although not all of these results were specific to colon cancers, these findings indicated that the activation of cGAS–STING signaling might promote the progression of cancers.

**Figure 2:**
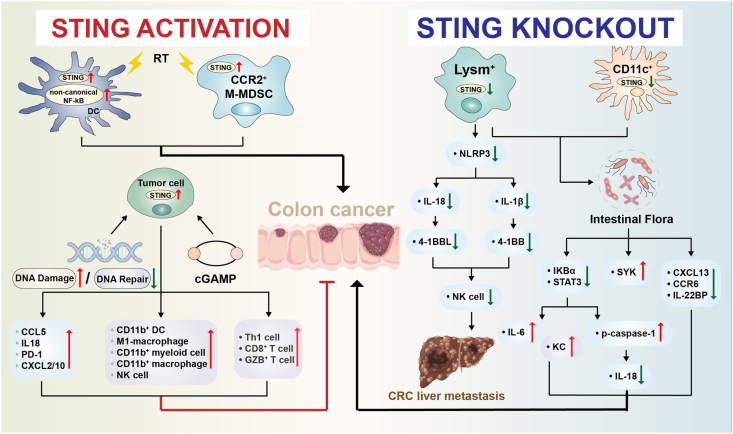
Bi-directional regulatory effects of the STING pathway in colon cancer development. STING activation in tumor cells suppresses colon cancer development, whereas STING deficiency in Lysm^+^ and CD11c^+^ cells exerts a pathogenic effect on the development of colon cancers by disturbing the balance of intestinal flora, suggesting the beneficial role of STING in the treatment of colon cancers. However, myeloid STING knockout before the occurrence of inflammation rather alleviates AOM/DSS-induced colon cancer, indicating the importance of the time point of myeloid STING deletion. Non-canonical NF-κB activation dependent on the STING pathway in dendritic cells (DC) and STING stimulation in monocytic-myeloid-derived suppressor cells (M-MDSC) results in tumor radiotherapy resistance, indicating a compensatory immunosuppressive effect in the radiotherapy of colon cancers.

Additionally, some reports have raised similar conclusions regarding the function of STING signaling in colon cancer, especially in response to radiotherapy (Fig. 2; Table 2). It was demonstrated that the activation of STING–type I IFN signaling in dendritic cells and CD8^+^ T cells hampered radiotherapy-induced anti-tumor effect by stimulating non-canonical NF-κB pathway.^51^ This suggested that STING-mediated type I IFN–NF-κB signaling played a detrimental role in the radiotherapy. Also, Liang and colleagues found that after localized ablative radiation in MC38 xenograft tumors, the STING–type I IFN pathway exerted a compensatory immunosuppressive effect by recruiting monocytic-myeloid-derived suppressor cells, and finally resulted in tumor radio-resistance.^52^ Further evidence is still required to elucidate the specific role of the STING pathway in radiotherapies against colon cancer, where genomic DNA suffers devastating damage and becomes a potential initiator of STING activation. Contradictorily, another study also focused on the potential effects of STING activation on myeloid-derived suppressor cells' function in colon cancer and suggested that partial inhibition of poly (ADP-ribose) polymerase (PARP) with a moderate dose of the PARP inhibitor, olaparib, inhibited AOM/DSS-induced colon cancer and MC38 tumor growth. This was achieved by inducing IRF3 phosphorylation and reversing the suppressive function of myeloid-derived suppressor cells, which was not strictly dependent on STING but totally independent of DNA damage.^53^

**Table 2:** The effects of STING on the progression of colon cancer.

Number	The function on STING	Mouse model	Triggers	Downstream signaling	Target cell	Reference
*Aggravate colon cancer*						
1	STING-mutant	AOM/DSS-induced colon cancer	/	NF-κB/STAT3	/	36
2	STING knockout	AOM/DSS-induced colon cancer	/	IL-18/IL-22BP	/	37
3	STING knockout	AOM/DSS-induced colon cancer	/	Sky	/	40
4	Lysm^+^ cell-specific STING knockout	AOM/DSS-induced colon cancer	/	/	Lysm^+^ cell	20
5	Myeloid-specific STING knockout mice	Colon cancer liver metastatic	/	IL-18/4-1BBL & IL-1β/4-1BB	Myeloid cell	31
6	STING activation	Ionizing radiation	/	TBK1/IRF3 & NF-κB/IFN-β	Dendritic cell	51
7	STING activation	MC38 xenograft tumors	/	CCR2 and M-MDSC infiltration	/	52
8	STING knockout	MC38 allograft colon cancer model	Loss of DNA-binding protein	/	/	55
*Alleviate colon cancer*						
1	Constitutive activation of STING	Injection with MC38 cells	/	/	/	42
2	Lysm^+^ cell- and Cd11c^+^ cell-specific STING knockout	AOM/DSS-induced colon cancer	*Helicobacter* spp	/	Lysm^+^ cell and Cd11c^+^ cell	21
3	STING activation	AOM/DSS- and syngeneic MC-38 cell-induced colon cancer	/	IRF3 and inhibition of MDSC function	/	53
4	STING activation	Syngeneic MC-38 cell-induced colon cancer	DNA damage repair deficiency/damaged DNA accumulation	IFN-α/β	Tumor cell	42
5	STING activation	Injection with MC38 cells	Absence of mismatch repair	IFN/CCL5 & CXCL10	Tumor cell	54
6	STING activation	Injection with CT26 cells	Inhibition of DNA damage repair	Infiltration of CD8^+^ T lymphocytes	Tumor cell	55

Note: DSS, dextran sulfate; M-MDSC, monocytic-myeloid-derived suppressor cells; AOM, azoxymethane.

## Tracing the origins of STING activation in colon cancer

DNA damage repair deficiency/damaged DNA accumulation and intestinal microbiota, as well as their products, have been investigated as endogenous and exogenous signal sources for the STING activation during the progression of colon cancer, respectively.^52^ Both AOM and its precursor, 1,2-dimethylhydrazine (DMH), can trigger DNA damage responses and thus become potential triggers of STING activation. It has been suggested that the expression of STING is notably enhanced in colonic tumor tissues of colon cancer mice after the treatment with AOM and DMH, which can trigger DNA damage via disturbing the mismatch repair of DNA.^37^ The absence of mismatch repair (MMR) genes, including MutL homolog 1 (MLH1) and MutS homolog 2 (MSH2), results in DNA damage and functions as endogenous signal sources for the STING activation. Vornholz et al found that in MMR-deficient tumors established by inoculation of MLH1/MSH2-depleted MC38 cells, the leakage of damaged DNA fragments triggered cGAS- and STING-mediated IFN-α/β production. Cancer cells expressing STING N153S exhibited increased expression of cytotoxic effector chemokines and molecules, such as Ccl5, Cxcl10, Gzmb, and IFN-γ, which contributed to an anti-tumor effect.^42^ Mowat et al also reported that the recruitment of CD8^+^ tumor-infiltrating lymphocytes and the production of Ccl5 and Cxcl10, as well as the endogenous activation of cGAS–STING–IFN signaling, were observed in MMR-deficient colon cancers. Similar results were demonstrated in organoids from colon cancer patients with MLH1 knockdown using lentiviral transduction.^54^ In addition to direct DNA damage, the loss of DNA-binding proteins also contributes to aberrant chromatin organization and abnormal DNA repair. Recently, in the MC38 allograft colon cancer model, deficiency of tumor-derived barrier-to-autointegration factor 1 (BANF1), a DNA-binding protein, was found to decrease tumor growth and promote infiltration of effector CD8^+^ T cells via activating IFN family-related innate immune responses. As expected, knockout of both cGAS and STING completely reversed the suppressive effects on tumor formation mediated by BANF1 depletion, demonstrating that the anti-tumor immune signature induced by BANF1 knockout depends on stimulation of the cGAS–STING pathway.^55^ Either cGAS and STING deletion rendered many human colorectal adenocarcinoma cells more vulnerable to oncolytic viral infection by suppressing DNA damage-induced cytokine production. These findings further suggested that MMR deficiency/DNA damage aggravation stimulated cGAS–STING signaling to increase chemokine production and create a “hot” tumor microenvironment for cytotoxic lymphocyte recruitment, and impaired STING responses enabled colon cancer cells to evade the host immunosurveillance process.^41^ Based on these findings, it is reasonable to speculate that additional targets and drugs that induce DNA damage responses can potentially synergize with cGAS–STING activation to achieve a superior therapeutic effect against colon cancer. For example, the deletion of PARP1, a DNA single-strand break repair protein, could induce DNA damage and activate the STING–IFN pathway in HCT116 cells.^56^ The chemotherapeutic drug, CPT-11, could also inhibit the process of colon cancer through stimulating dsDNA break and the corresponding cGAS–STING pathway.^57^^,^^58^ Correspondingly, Src homology-2 domain-containing protein tyrosine phosphatase-2 (SHP2) suppressed DNA damage repair and activated the STING pathway via dephosphorylating PARP1 at Tyr 907 in CPT-11-treated HCT116 cells. SHP2 agonist lovastatin co-treated with CPT-11 inhibited CT26 tumor growth by facilitating DNA damage aggravation and STING-mediated infiltration of CD8^+^ T lymphocytes.^58^

From another perspective, studies have revealed that, in colon cancer, the diversity and abundance of intestinal bacterial species influence the therapeutic outcome of immunotherapies, in which the STING pathway is involved.^60^^,^^61^ At the level of genus, *Akkermansia*, *Ligilactobacillus*, and *Subdoligranulum* increased the most in colorectal cancer patients, while *Bacteroides* and *Dialister* decreased.^59^ The antibiotic treatment in mice with colon cancer inhibited the activation of the STING pathway and promoted tumor development. Ahn et al reported that in the *Helicobacter* spp positive barrier environment, more polyps in colon tissue were developed in STING knockout mice after AOM/DSS induction, when compared with WT mice. However, in *Helicobacter* spp negative housing conditions, STING-deficient mice did not show a higher number of polyp formation, alluding to the importance of the STING-dependent mechanism in preventing intestinal flora-related carcinogenesis. Furthermore, they demonstrated that the absence of cGAS did not facilitate polyp formation, further indicating that bacterial products, rather than genomic or damaged DNA, are primary causal factors of STING-mediated anti-tumor effects.^21^ Interestingly, the enrichment of intestinal dominant bacteria in the tumor microenvironment, rather than the intestine, could also enhance the efficacy of immunotherapy against colon cancer.^62^ Specifically, the local delivery of *Bifidobacterium* potently stimulated STING signaling at tumor sites, rather than regulating gut immunity, increasing type I IFN signaling, and cross-priming tumor-associated dendritic cells. The above studies inspire us to explore new therapeutic strategies for inducing STING-dependent anti-tumor effects from the perspective of microbiota or their related products. Moreover, further investigation is needed to gain a more comprehensive understanding of the specific biological trigger of the STING pathway during the tumorigenesis of colon cancers.

## Three determinants underlying the paradoxical functions of the STING pathway

**Figure 3:**
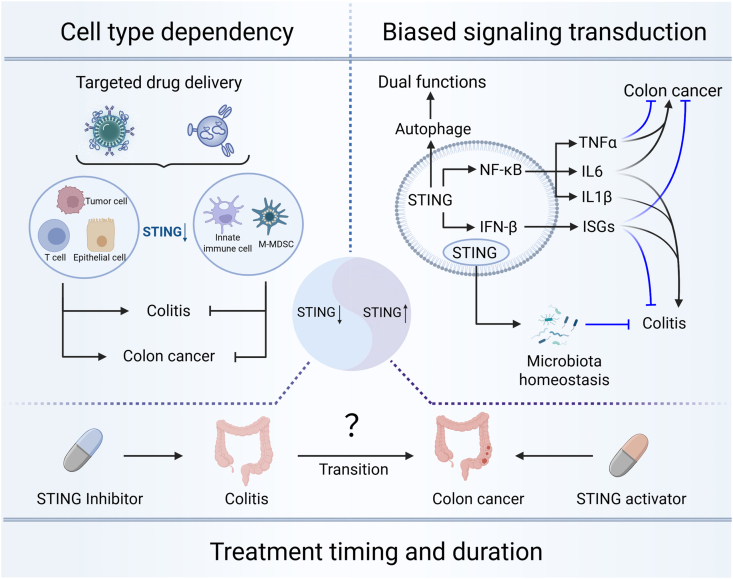
Three determinants underlying the paradoxical functions of the STING pathway. Three determinants include cell type dependency, treatment timing and duration, and biased signaling transduction. These factors contribute to the bidirectional roles of STING in colitis and colon cancer.

The bidirectional roles of STING in the same disease or at different disease stages can be attributed to the following three major reasons (Fig. 3): i) cell type dependency; ii) treatment timing and duration; iii) biased signaling transduction.

Firstly, we have summarized the distinct functions of STING in epithelial cells, innate immune cells, adaptive immune cells, and tumor cells. Activation of STING in innate immune cells exacerbates intestinal inflammation, whereas its activation in intestinal epithelial cells impairs the antibacterial capacity of these cells and causes severe damage to the intestinal barrier. Currently, targeted drug delivery that directly leads to drug delivery to myeloid cells, epithelial cells, and T cells has been achieved. Li et al reported a novel microbubble-assisted ultrasound-guided immunotherapy of cancer (MUSIC) strategy, which can specifically target antigen-presenting cells for effective delivery of STING activators.^63^ EVs have been used to specifically deliver biopharmaceuticals to T cells,^64^ while butyrate-modified liposomes encapsulating small-molecule compounds can specifically target intestinal epithelial cells. The application of these novel targeted delivery technologies enables the manipulation of STING signaling in specific cell types, which is expected to achieve better therapeutic efficacy with fewer side effects.

Secondly, the timing and duration of STING modulation matter. Before the treatment, we propose accurately distinguishing the stage of the disease. Depending on whether the disease is in the cancer-free inflammatory stage, established cancer stage, or ongoing cancer stage, the appropriate inhibitors or agonists should be selected accordingly. Furthermore, during the treatment process, it is necessary to control the frequency and intensity of drug administration to avoid side effects that impair therapeutic efficacy, such as excessive infection or T cell exhaustion. What is even more noteworthy, yet has not been well-studied, is the crucial role STING plays in the stage of transformation from colitis to colon cancer. DNA damage response genes mark the early transition from colitis to neoplasia in colitis-associated colon cancer. Under chronic inflammation conditions, microsatellite instability is caused by the early loss of DNA damage response (DDR) genes, and these pathogenic changes initiate dysplasia, ultimately leading to the formation of colon cancers.^65^ When reduced expression of Dicer is restored in inflamed colon tissues, STING signaling is suppressed, resulting in improved colitis and delayed tumor development.^66^ In the AOM/DSS-induced colon cancer mouse model, treatment with palbociclib and H-151 from the first day of modeling or early myeloid STING deficiency before inflammation could delay tumor onset and hinder tumor development by alleviating excessive colonic inflammation.^20^^,^^67^ Previous studies have pointed out that other inflammation-driven carcinogenesis is mediated through STING, as evidenced by the finding that WT mice adoptively transferred with *STING*^*−/−*^ bone marrow are almost completely resistant to inflammation-induced skin carcinogenesis.^68^ Mechanistically, STING-dependent hyper-inflammation triggered by excessive DNA damage, in turn, amplifies mutational burden through reactive oxygen and nitrogen species generation and the disruption of DNA-repair machinery, establishing a self-perpetuating cycle that drives oncogenic cell transformation.^69^ Furthermore, recent studies have found that the leakage of intracellular nuclear dsDNA or mitochondrial dsDNA can activate STING and induce cellular senescence,^70^^,^^71^ which is also a trigger for tumor progression.^72^ This suggests that STING may participate in the regulation of cell fate and tumorigenesis through other non-inflammation-dependent pathways, such as directly regulating p53 and p21. Thus, targeting STING during the inflammation-carcinogenesis stage represents a promising direction worthy of exploration, with significant implications for clinical intervention strategies.

Thirdly, although research on STING’s biased signaling transduction remains limited, we regard it as highly significant. There are multiple downstream signaling pathways of STING, each of which can elicit distinct responses, resulting in distinct roles in disease. Specifically, as a classical antiviral signaling pathway, STING regulates IFN-β expression by promoting IRF3 nuclear translocation, which in turn induces ISGs^73^ and chemokines to regulate T cell-dependent adaptive immune responses to favor the development of colitis.^74^ Greta et al reported that type I IFNs, including IFN-β, could directly inhibit myeloid cell-mediated inflammation via the STAT1/NLRP1/3 pathway, exerting potential therapeutic effects against colitis.^75^ Furthermore, STING activation could induce IKKε phosphorylation, driving NF-κB signaling transduction, which then diverge into several pathways that could differentially regulate the pathogenesis of colitis and colitis-associated colon cancer: i) IL-1β-mediated pyroptosis and DAMP release to promote colitis^76^; ii) IL-6 expression to promote colitis and tumor cell growth^77^; iii) TNF-α secretion and chemokine release to deliver anti-tumor effects by remodeling the tumor microenvironment.^45^ These dual-directional downstream effects collectively contribute to the paradoxical functions of the STING pathway. Moreover, nuclear STING and cytoplasmic STING could exhibit distinct functions as described above. Nuclear STING regulates microbiota homeostasis via a non-canonical pathway (STING/PML/AHR) and exerts a protective effect in colitis. In contrast, cytoplasmic STING promotes colitis progression via the classical pro-inflammatory pathway (cGAS/STING/IRF3/NF-κB). Thus, clarifying which downstream pathways are activated by STING in a specific context would be of great interest. Recent studies have further indicated that STING may possess more conserved biological functions beyond activating innate immune responses, such as acting as a proton channel and inducing autophagy.^78^^,^^79^ These newly discovered functions are highly relevant to cell fate and inflammatory responses, suggesting that the actual roles of STING in colitis and colon cancer are even more complex, making it difficult to predict if different STING agonists or inhibitors will have consistent biological effects on colitis or colon cancer. Although we have systematically reviewed current STING pathway modulators under investigation (Table 3), none have yet yielded satisfactory clinical outcomes. We believe the biased signaling transduction might be the possible reason.

**Table 3:** Research stages of the STING inhibitors/agonists entering clinical trials for the treatment of colitis and colon cancer.

Name	STING-specific	Effect on STING	Current status	Subject	Samples	Latest results	Applications in colitis/colon cancer	Reference
MK-1454	Yes	↑	FDA approval	Advanced/metastatic solid tumors or lymphomas	156	Completed	Experimental colon cancer	84
TAK-676	Yes	↑	FDA approval	Non-small-cell lung cancer, triple-negative breast cancer, and squamous-cell carcinoma of the head and neck	34	Completed	/	/
α-Mangostin	Yes	↑	FDA disapprove	Recurrent aphthous stomatitis	48	Recruiting	Experimental colon cancer	85
DMXAA (ASA404)	Yes	↑	/	Refractory tumors	15	Completed	Experimental colon cancer	86
CRD3874-SI	Yes	↑	FDA approval	Acute myeloid leukemia; advanced/metastatic malignant solid tumors	36; 72	Recruiting	/	/
[68Ga]Ga-Sa-DABI-4	Yes	↑	FDA disapprove	Cancer patients	30	Recruiting	/	/
Microparticles delivered cGAMP	Yes	↑	FDA disapprove	Relapsing remitting multiple sclerosis	40	Unknown	/	/
E7766	Yes	↑	FDA approval	Advanced solid tumors or lymphomas	24	Terminated	/	/
PULSAR-ICI + IMSA101	Yes	↑	FDA approval	Oligometastatic non-small cell lung cancer and renal cell carcinoma; oligoprogressive solid tumor malignancies; metastatic kidney cancer	6; 16; 15	Terminated	/	/
MIW815 (ADU-S100) + PDR001	Yes	↑	FDA approval	Advanced/metastatic solid tumors or lymphomas	106	Terminated	/	/
SNX281	Yes	↑	FDA approval	Advanced solid tumors and lymphoma	27	Terminated	/	/
Suramin	Yes	↓	/	Hormone-refractory prostate cancer	390	Completed	Experimental colitis	87
Palbociclib	Yes	↓	FDA disapprove	Locally advanced and/or metastatic breast cancer	815	Completed	Experimental colitis	88

Elucidating the bias in STING signal transduction from a structural perspective will be a breakthrough for future development of STING-based therapeutic strategies. However, deciphering its binding modes with upstream signaling molecules as an adaptor, its trans-organelle trafficking patterns during activation, and its selectivity in recruiting downstream factors could be challenging via conventional biological approaches. AlphaFold is a highly efficient tool that can efficiently predict the conformational changes of STING proteins, aiding us in gaining structural insights.^80^ As reported, STING oligomerization and phase-separating properties determined the downstream signaling transduction of STING.^81^ Recent studies have found that AlphaFold can predict homo-oligomeric assemblies and phase-separation states.^82^^,^^83^ Full analysis of the critical phase-separation sites and oligomerization sites of STING may also be achievable, which will aid in the development of targeted drug candidates. Additionally, the structure of the selective STING signaling transduction complex can also be defined with the help of AlphaFold. AlphaFold-Multimer can predict the potential binding interface between STING and downstream molecules (such as TBK1, IRF3, and IKKε), aiding in the identification of druggable pockets with “pro-inflammatory/anti-tumor” specificity for further virtual drug screening. Moreover, with the advances in structure-guided biological assays, such as Flash BRET technology,^73^ we anticipate that next-generation STING modulators endowed with downstream-signaling selectivity will soon be developed to enhance therapeutic efficacy while averting unpredictable adverse events.

## Conclusion

In summary, STING pathway plays distinct functions in colitis and colon cancer as well as in different types of immune cells. A deeper understanding of the STING signaling pathway indicates that developing cell-specific and downstream-selective STING modulators, in cooperation with precise timing and treatment duration, holds significant therapeutic potential against colitis and colon cancer.

## CRediT authorship contribution statement

**Jiaorong Qu:** Writing – original draft, Supervision, Methodology, Investigation. **Yajie Cai:** Writing – original draft, Visualization, Methodology, Investigation. **Fanghong Li:** Visualization, Validation. **Yufei Li:** Validation, Methodology. **Runping Liu:** Writing – review & editing, Supervision, Conceptualization.

## Funding

This work was supported by grants from the Excellent Young Scientists Fund from the 10.13039/501100001809National Natural Science Foundation of China (No. 82322075 to R.L.), the 10.13039/501100012166National Key Research and Development Program on Modernization of Traditional Chinese Medicine (China) (No. 2022YFC3502104 to R.L.), the 10.13039/501100001809National Natural Science Foundation of China (No. 82404984 to J.Q.), and the 10.13039/501100002338Fundamental Research Funds for the Central Universities (China) (No. 2024-JYB-JBZD-055 to J.Q.).

## Conflict of interests

The authors declared no conflict of interests.
